# Current strategies for the management of valuable compounds from hops waste for a circular economy

**DOI:** 10.1016/j.fochx.2023.100876

**Published:** 2023-09-13

**Authors:** Liana Claudia Salanță, Anca Corina Fărcaş, Andrei Borșa, Carmen Rodica Pop

**Affiliations:** a Department of Food Science, Faculty of Food Science and Technology, University of Agricultural Sciences and Veterinary Medicine Cluj-Napoca, 400372 Cluj-Napoca, Romania; b Department of Food Engineering, Faculty of Food Science and Technology, University of Agricultural Sciences and Veterinary Medicine Cluj-Napoca, 400372 Cluj-Napoca, Romania

**Keywords:** Brewery waste, Spent hops, Trub, Bioactive compounds, Sustainability

## Abstract

World beer production generates large volumes of waste discharged with every brew. Recently, new methods of reducing and reusing hops waste: hot trub (HT), and brewer-spent hops (BSH) are being exploited to improve the circular economy processes. This review outlines the current achievements in the management of hops waste. Following an in-depth review of various scientific publications, current strategies are discussed as a sustainable alternative to food waste exploitation and an inexpensive source of valuable compounds. Moreover, key aspects concerning the nutritional value of hops waste and the potential to enhance the functional properties of food and beverages are highlighted. Due to their nutritional composition, hops residues may be used as prospective sources of added-value co-products or additives for food enrichment, especially for products rich in fat, or as a new source of vegetable protein.

## Highlights

• This research outlines the current achievements in the management of hops waste.
• Hops by-products represent a sustainable source of valuable bioactive compounds.
• Strategies for reusing hops waste are being applied to create new products.
• Spent hops exploitation yields nutritional, economic, and environmental advantages.

## 1 Introduction

For centuries the female inflorescence of the hop plant (*Humulus lupulus* L. Cannabaceae) has been one of the main beer ingredients imparting flavor and bitterness (Salanţă et al., 2015). The chemical composition has been widely explored, and to date, scientists have focused their research on hop acids, essential oils, and flavonoids (Bartmańska et al., 2018, Cvijetić et al., 2021, Duarte et al., 2022, Salanţă et al., 2016). Also, the beneficial and pharmacological properties of compounds isolated from the hop have been described over the years (Bolton et al., 2020, Karabín and Hudcov, 2016, Olsovska et al., 2016, Zanoli and Zavatti, 2008). Hops are responsible for beer’s bitterness and aroma due to the hop’s α-acids that isomerize during the wort boiling process. Rich in essential oils, flavonoids, tannins, and minerals (magnesium, manganese, iron, zinc) (Poreda et al., 2015), hops can also have antioxidative capacity especially when single hop dosage is exchanged with incremental hop dosage, dry hopping, or the use of a pre-isomerized hop by-product during the beer production process (Kunz et al., 2014). Hop compounds show biological activities such as antioxidant, antimicrobial, antifungal, antiviral, anti-inflammatory, and anticancer and can be used in the food and health industry (Astray et al., 2020).

**Fig. 1:**
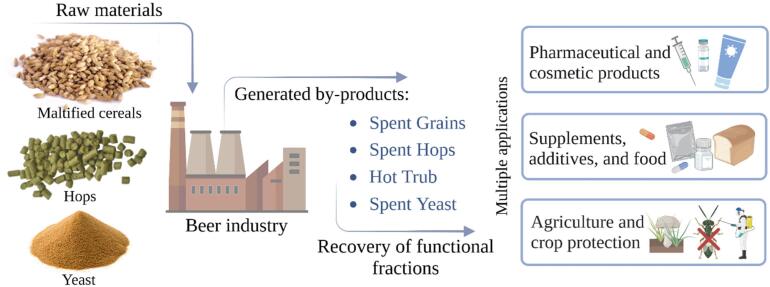
Brewing wastes and further applications.

The global Beer Market is valued at USD 768.55 Billion in the year 2022 and is forecast to reach a value of USD 996.49 Billion by the year 2030. The Global Market is projected to grow exhibiting a Compound Annual Growth Rate (CAGR) of 3.30% over the forecast period. Considering that, the path to sustainability in the brewing sector has significant potential due to the large volumes of waste discharged with every brew (Hejna, 2022). During the production of beer, the main wastes are brewer-spent grain (BSG), hot trub (HT), and brewer-spent hops (BSH), along with the yeast (BSY) after fermentation. Fig. 1 represents the most valuable by-products of brewing (Rachwał et al., 2020).

Brewery wastes can negatively impact the environment due to their high nutritional value, so their use has been researched and various applications have been found. Beer by-products can be used in agriculture as animal feed, in the food industry as insect repellent or protein, mineral, vitamin, or polyphenol food supplementation, as a substrate for composting, microbial or microalgae growth, and as biofuel rich in methane and hydrogen (Karlović et al., 2020). The environmental impact of brewery waste is measured in a series of parameters: pH, temperature, total suspended solids (TSS), chemical oxygen demand (COD), and biological oxygen demand (BOD) (APHA (American Public Health Association), 2017). Devolli et al., 2018, optimized the brewing process to lower the organic matter brewery wastes. After optimization, processing trub and spent grain wastewater had a BOD/COD ratio of 65,66% meaning that the organic compounds in the wastewater are easily biodegradable (Devolli et al., 2018). In this regard, the main purpose of this review is to provide updated information related to recent developments in the management of hops waste. This bibliographic research utilized a narrative review methodology to synthesize existing evidence on the potential applications of hops by-products. Relevant studies published in the past decade were selected based on their quality, data availability, and relevance to the research topic. Academic databases, including PubMed, Science Direct, Scopus, and Web of Science, were consulted to retrieve relevant studies, while grey literature sources were excluded. The cited studies were selected based on their relevance to the research topic and the quality of their design and methodology. To conduct the search, a combination of keywords such as “hops”, “*Humulus lupulus* L. ”, “by-products”, “waste”, “circular economy”, “bioactive compounds”, “antioxidants”, “xanthohumol”, “chalcone”, and “health benefits” were utilized, along with Boolean operators.

## 2 Importance and chemical composition of spent hops

The BSH represents the residue from hops used in the brewing industry; only 15% of the hops constituents will be transferred into the beer, whereas 85% will become a by-product, BSH material and they can be discarded together with HT (Bedini et al., 2015). The HT (brewing waste) is a precipitation product of the wort boiling process that includes insoluble hop materials, condensation products of hop polyphenols and wort proteins, and isomerized hop acids adsorbed on the HT solids (Fărcaş et al., 2017). HT is characterized by high moisture (80–90%), high molar-mass protein content (almost 50%), and over 20% of reducing sugar concentration which contributes to high carbon concentration but a low C/N ratio that allows its use as a fermentation additive (Santos Mathias et al., 2015). It is obtained after boiling the wort and it has important nutrients (proteins and carbohydrates) and compounds with biological activity (antioxidants). The main challenge in food industrial use of this byproduct is managing bitterness conferred by isomerized α-acids (cohumulone, humulone, adhumulone) and polyphenols (catechin and epicatechin) (Habschied et al., 2021).

The HT composition and yield are affected by several factors, such as raw materials (type of barley and hops; concentration and degree of solubilization of substances from hops; proportion and types of adjuncts used; malt and/or adjuncts in the drying process and type of milling (particle size and shape)) and brewing process characteristics (pH control; medium concentration of ions and polyphenols; homogenization time and oxidation during boiling stage) (Senna Ferreira Costa et al., 2021). Such waste material is usually disposed of in agricultural fields, as a fertilizer and soil conditioner, due to the high nitrogen content or mixed with spent grain and sent to animal feeding.

BSH is characterized by a higher proportion of fiber (22–23%), essential oils, lipids (4.5%), proteins (22–23%), and minerals (6–6.5%). Compared with the other waste BSH recovery is becoming increasingly important due to the essential oil content, mainly composed of sesquiterpene hydrocarbons (37%), monoterpene hydrocarbons (27%), non-terpene derivatives (18%), oxygenated sesquiterpenes (8%) and oxygenated monoterpenes (4%) (Amoriello and Ciccoritti, 2021, Karlović et al., 2020). Thus, by re-using the high-value compounds from BSH, using innovative and unconventional ways, the “zero-waste” desiderate can be reached, which is essential in a circular economy.

Rheological and microbiological properties of BSH and HT were studied by Sterczynska et al., 2021 on different hop varieties. The wort boiling time and the malt type affected the maximum viscosity value. The longer boiling precipitated sediment was found to be the most convenient material for the brewing industry because of its low viscosity which makes the composting process cheaper and easier. Hop sediments weren’t microbiologically contaminated and remained sterile so they could be further used (Sterczyńska et al., 2021).

HT contains relevant nutrients, such as proteins, carbohydrates, and phytochemical compounds, but its applications in the food segment are limited by its bitter flavor (Saraiva et al., 2019). The bitterness and astringency mainly come from the phenolic compounds (tannins) of the hops (Habschied et al., 2021). Recently, Saraiva et al., 2019, focused on reducing the bitter compounds in the HT while maintaining nutritional and functional properties. In their study, the protein content was concentrated, and the bitterness and water absorption were reduced.

In recent years, several attempts have been made to address the utilization of BSH as a natural repellent in the protection of stored food against insects (Bedini et al., 2015), in medicine and cosmetology (Kerby et al., 2017), as a source of protein for the food industry (Saraiva et al., 2019), as a valuable resource for biotechnological applications (Radosavljević et al., 2019; Senna Ferreira Costa et al., 2021) or as a valuable compost (Kopeć et al., 2022).

The craft brewing industry in many countries has recently undergone a rapid expansion in the number of breweries. The growth of the beer market is primarily driven by the increasing demand for premium and craft beers (Salanță et al., 2020). In addition, the increasing popularity of home-brewing and microbreweries is anticipated to contribute to market expansion over the forecast period. Additionally, the industry is marked by high fixed costs, low marginal costs, and a limited number of brands. Brewers must continue to innovate and invest in marketing and product development to remain competitive.

The production of 1 hL of beer generates 0.2–0.4 kg of BHS. Microbreweries use about 10 times more quantities of hops per liter when compared with the traditional big beer companies (approx. 100 g hop cones/pellets/1hL beer) (Machado et al., 2019), leading to increasing quantities of BSH. Almost always, the BSH is discarded after a single use, although they are still rich in bioactive compounds. The estimated amount of filtration sediments generated by the brewing industry (waste code 020780) ranges between 0.55 and 2.61 kg·hL^−1^ of beer and totals 1.22 kg·hL^−1^ on average. Filtration sediments are considered to be the largest source of waste in a brewery (17.6 kg·hL^−1^), as on average they amount to 7% of the brewer’s grain mass. Considering that in 2022, small and independent brewers collectively produced more than 24.3 million barrels of beer just in the US alone, according to The Brewers Association, with some brewers hopping at rates of 1 kg/hL or dry hopping at rates up to 2 kg/hL (5 lb/barrel), considerable waste is generated in terms of both beer loss and spent hops (Hauser et al., 2019). Considering the scale of beer consumption there is the possibility of local environmental and economic problems. Craft brewers explore various unique and sustainable methods of by-product disposal to benefit both the environment and their breweries’ economic output.

Regarding the financial aspect, this massive quantity of hops, and the prices reaching 20 Euro per kilo, make it one of the most expensive ingredients in beer manufacturing, typically comprising over fifty percent of the budget for hop-heavy recipes. One more reason to identify solutions in a circular system. Approximately one-third of the dry matter composition of hops is lost during dry-hopping regardless of hop variety, indicating that spent dry-hops contain considerable brewing value and have the potential for re-use (Hauser et al., 2019)by reducing the amount of hops used as well as the final amounts of SH and at the same time increasing profitability.

With the rising popularity of the specialty beer segment (Salanță et al., 2020), dry hopping has emerged as a ubiquitous technique for achieving pronounced hop aroma and flavor in beer. Given the growth of nonalcoholic beer (NAB) and alcoholic-free beer (AFB), BSH could be utilized as a flavoring agent in the beer industry. There are primarily two options for de-alcoholizing beer, mainly used by craft brewers: evaporation of ethanol by heat treatment, which also removes desired aroma-active compounds, and stopping fermentation, which results in beers with high levels of unfermented sugar and lower levels of aroma-active fermentation products like 3-methyl-1-butanol or 3-methyl butyl acetate (Salanță et al., 2020). In the study made by Brendel et al., 2020, hop-derived odorants (linalool, geraniol, myrcene, and esters), transferred in rates between 20 and 90% in AFB, also inducing the formation of ethyl esters of hop-derived monocarboxylic acids. Consequently, dry hopping may be an option for compensating for these aroma deficiencies (Brendel et al., 2020).

Taking into consideration the low temperatures at which dry-hopping is carried out (relative to kettle additions), hop-derived bitterness precursors are neither isomerized nor transferred to beer and remain, in significant quantities, within the discarded waste material (Hauser et al., 2019). Hauser et al., 2019, carried out a study to assess whether spent dry hops could potentially be re-used for bittering. The results demonstrated that from both an in-brewery utilization and organoleptic perspective, spent dry-hops could provide a feasible alternative to traditional kettle additions, while potentially saving brewers money and reducing environmental impact.

## 3 Extraction of value-added compounds from spent hops

*Phenolic compounds.* Hop flowers have 4–14% dry-weight phenolic compounds, mainly phenolic acids, chalcones, flavonoids, and proanthocyanidins, and various extraction methods are used to recover these molecules: solid–liquid extraction with solvents of different polarities, supercritical CO_2_ extraction, deep eutectic solvents extraction (Silva et al., 2023). Gandolpho et al., 2021, optimized ultrasound-assisted extraction of phenolic compounds from HT, obtaining 7.23 mg of gallic acid/g HT, during 30 min of extraction at 36 °C, with a solid: liquid ratio of 1 g/32 ml and 58% ethanol as solvent (Gandolpho et al., 2021). Deep eutectic solvents can be used for the simultaneous extraction of xanthohumol(Xn) and proteins from BSH. Depending on the chosen solvent, the Xn extraction yield was 0.52–1.92 mg/g BSH and the protein content varied between 40 and 64%, being a potential source for nutraceuticals and functional foods (Grudniewska & Pastyrczyk, 2022).

*Xanthohumol and derivatives.* BSH is rich in prenylated flavonoids with biological activities such as xanthohumol (Jackowski et al., 2015). Breweries try to enhance xanthohumol concentrations in beer to offer functional beers to consumers, but the main challenge is the low solubility in water. Different strategies like using special hop products, the addition of xanthohumol extracts, optimization of hop addition, or processing hop wort at high temperatures (60 °C) improved the solubility of Xn and increased its content (Karabín et al., 2013). Grudniewska et al., 2020, used four choline-chloride-based deep eutectic solvents as a simple and green method to extract xanthohumol. The highest extraction yield (2.30 mg/g BSH) was obtained using choline-chloride and propylene-glycol with 5 % water as deep eutectic solvent (DES), a percent of 1:50 BSH/DES (w/w), and 3:1 antisolvent/DES (v/w) at 60 °C for 60 min (Grudniewska & Popłoński, 2020). Xanthohumol possesses numerous biological properties: anticancer agent, acting as a chemoprotective and cytotoxic compound (Hrnčič et al., 2019), antimicrobial (on Gram-negative and positive bacteria but also on fungi and yeasts) (Stompor & Zarowska, 2016), phytoestrogen, modulating hormonal imbalance in menopausal women (Sandoval-Ramírez et al., 2017), sedative, binding to GABA_A_ receptors (Karabín & Hudcov, 2016), anti-inflammatory (an effect that can be enhanced by micellar solubilization) (Khayyal et al., 2020), and apoptotic, directly interacting with mitochondrial electron transfer chain complex (Zhang et al., 2015). Humulone, lupulone, and xanthohumol exerted antibiofilm properties against methicillin-susceptible, and resistant strains of Staphylococcus sp. with potential medical applications: protection of intravenous and urinary catheters, prosthetic joints, and artificial heart valves (Bogdanova et al., 2018). Xanthohumol showed antimicrobial activity upon six bacterial strains that develop biofilms over dental implant surfaces: *Streptococcus oralis, Actinomyces naeslundii, Veillonella parvula, Fusobacterium nucleatum, Porphyromonas gingivalis* and *Aggregatibacter actinomycetemcomitans* and 100 µM extract could be used in prevention and treatment of *peri*-implantitis (Alonso-Español et al., 2023). Xanthohumol has a selective anti-inflammatory effect on cement oblasts which may be useful in the modulation of inflammation during orthodontic therapy (Niederau et al., 2022).

*Flavonoids.* The 8-prenylnaringenin and its 8-geranyl derivative are hop flavonoids with apoptotic activity in the human Burkitt lymphoma cell line and inhibition of voltage-gated potassium channels from human leukemic T cell line and could be used as natural compounds for leukemia treatment (Menezes et al., 2016). Calvo-Castro et al., 2018, compared oral bioavailability and safety of 6- prenylnaringenin (6-PN) and 8-prenylnaringenin (8-PN) in a double-blind randomized trial, 8-PN having better bioavailability than its isomer (4–5 times higher) with immunostimulatory activity (Calvo-Castro et al., 2018). The 8-PN was obtained by bioconversion of isoxanthohumol(IX) from BSH using resting cells of *Eubacterium limosum*. BSH prenylated flavonoids have antimicrobial properties so the bacteria were used at the resting phase, at a pH of 7.8–8, and in a reinforced clostridial medium for proper enzymatic induction and maximal solubility (Moens et al., 2020). Standardized extracts in 6-PN, 8-PN, IX, and XN of spent hops were administered to post-menopausal women and their pharmacokinetics was evaluated. With a good half-life and low toxicity, a single daily dose could assure a natural-source hormone replacement therapy for menopausal symptoms (Breemen et al., 2014).

*Essential oils.* Cibaka et al., 2016, characterized essential oils found in three hop varieties and found a grapefruit-like 3-sulfanyl-4-methyl pentane-1-ol, a polyfunctional thiol as a common compound. Glutathione S-conjugates were the major fraction of polyfunctional thiols in hops with various tastes, offering a preview of the sensorial profile of beer made from these hop varieties (Kankolongo Cibaka et al., 2016). Hops essential oils are effective against two invasive species: *Aedes albopictus* (Asian tiger mosquito) and *Physella acuta* (freshwater bladder snail) and could be used as cheap and environmentally friendly insecticides and molluscicides (Bedini et al., 2016).

*Bitter acids: α and β-acids.* Hops β-acids combined with lipopolysaccharides exerted anti-inflammatory activity, decreasing the expression of IL-1β, a proinflammatory cytokine (Bortoluzzi et al., 2016). Xu et al., 2021 developed a chitosan–gelatin edible film enriched with hops extract, hop α and β-acids which showed good UV-light barrier properties and antioxidant activities, being a promising safe, effective, and environmentally friendly food packaging alternative, even for fruits and vegetables with post-harvesting rips (Xu et al., 2021). Purified extracts isolated from hop α and β-acids and xanthohumol were tested for inhibitory effect against *Clostridium difficile*, a bacterium that causes nosocomial gastrointestinal infections in humans. After two days of application, fecal bacterial load was significantly decreased, xanthohumol being more potent than hops acids, having the advantage of low toxicity because of its minimal resorption in the intestine (Sleha et al., 2021). Cermak et al., 2017, tested the same three compounds’ activity on anaerobic gut bacteria and found supplementary antibacterial activity upon *Bacteroides fragilis* and *Clostridium perfringens* (Cermak et al., 2017)*.*

*Proteins.* HT was used as a protein source, with an extraction yield of 19.89%, at a pH of 12.31, and an extraction temperature of 80℃ for 51.78 min. The protein isolate was rich in phenolic compounds with antioxidant activity and could be used in functional food development (Saraiva et al., 2021). Lomuscio et al., 2022 fortified durum wheat fresh pasta with 5–15 g HT/100 g pasta, obtaining a high fiber and protein-enriched product that showed a lower glucose release. Pasta samples with 10% HT extract had the best overall acceptability (Lomuscio et al., 2022).

## 4 Solutions for industrial waste management of hops

Hops residues may be used as a substrate for the cultivation of microorganisms, organic fertilizer (given its carbon, nitrogen, and phosphorus content), extraction of various compounds of interest: polyphenols, flavonoids, protein concentrates or peptide source, and biofuel production (because of the anaerobic digestion made by microorganisms that generates methane that is further transformed into biogas) (Ruiz-Ruiz et al., 2019). The recent hops waste applications are presented in Table 1.

**Table 1:** The main applications of hops waste.

Use	Source	Process	Advantages	Disadvantages	Efficiency	Applicability	References
Biogas generator	Hops waste co-digested with grains	Anaerobic digestion at 35 °C (mesophilic) and 55 °C (thermophilic)	Thermophilic	Mesophilic	Mesophilic: 184 m^3^/day of biogas with 126.01 kW boiler fuel	Use of beer production wastes and heat to generate biogas that produces energy for a sustainable brewing process	(Siqueiros et al., 2019)
			12–14 days needed.	15–30 days needed.			
					Thermophilic: 163 m^3^/day of biogas with 76.48 kW boiler fuel		
			Better methanol extraction yields and quality -> higher energy contents.	Lower methanol extraction yields -> lower energy content			
Insect repellent	Spent hops (BSH)	Hydro-distillation of dried spent hops for extraction of essential oils (myrcene being the major constituent with repellent activity)	Low-cost resource	Low activity on *S. granarius*	Extraction yield of essential oils: 0.11% dry weight	Insect pest repellent on *R. dominica* and *S. granarius*	(Bedini et al., 2015)
			Eco-friendly insect repellent products suitable for the protection of stored food against *R. dominica*				
Stabilizer for emulsion gels	A mixture of trub (HT) and spent brewer yeast (BSY)	Byproduct was dried, powdered, dispersed in water, and added to the emulsion	↑rheological stability at higher pH and byproduct concentration	Decrease of luminosity when byproduct concentration increased	High concentration of byproduct at pH = 12 obtained stable high internal phase emulsion (HIPE) gels with homogeneously distributed oil droplets of regular size and polyhedral structure	HIPE gels stabilizers	(López-García et al., 2021)
Compost	HT and BSH	Composting carried for 90 days with maize straw	High concentrations and a large variety of microorganisms that inhabit the spent hops compost	Low concentration but a large variety of microorganisms that inhabit hot trub compost	Vegetative bacteria in SHcompost = 2478 x10^2^ CFU g^−1^ DM	Composting (BSH compost) or plant-growth retarder (HT compost)	(Kopeć et al., 2022)
					Vegetative bacteria in HTcompost 1532 x10^2^ CFU g^−1^ DM		
			No pathogens found				
Platelets antiaggregant	BSH extract	Supercritical CO_2_ extraction followed by drying and various treatments necessary to identify polyphenols and platelet reactivity	50% phenolic compounds content with antioxidant activity	Lack of xanthohumol due to chosen extraction process	Inhibition of 5′-adenosine diphosphate (ADP)-induced platelet aggregation up to 11% for 7.5 µg/ml or up to 23% for 15 µg/ml	Pharmaceutical industry as nutraceutical or herbal supplement with antiplatelet activity	(Luzak et al., 2016)
			Strong antiplatelet activity in vitro and proved its beneficial effects in vivo on rats				
		Administration of SHE to rats with induced diabetes					
Antioxidants and antimicrobials	HT	Ultrasound-assisted extraction. The extract was treated to obtain the hexane fraction (HF), ethyl acetate fraction (EAF), a butanoic fraction (BF), and aqueous fraction (AF)	EAF showed the highest antioxidant activity	No phenolic compounds or flavonoids in AF	Extraction yield: EAF (45.1%), HF (27.1%), BF (22.2%), AF (5.6%).	The pharmaceutical industry as a nutraceutical	(Senna Ferreira Costa et al., 2021)
			HF shows the highest antibacterial potential and cytotoxic activity	Molecular modifications that occurred during extraction reduced the antioxidant activity of compounds	Phenolic content: 8.237–13.390 mg GAE/g hot trub		
					Flavonoid content: 0.019–1.643 mg QE/g hot trub		
Beer production	BSH	Rinsed BSH with water and frozen (called recycled hops) and used afterward in beer production	Physicochemical properties maintained	Decrease with 30% of volatile compounds (isoalpha acids)	Phenolic compounds: 251.748–268.107 mg GAE/L	Using the same hops in two beer production processes	(Gasiński et al., 2022)
					IBU units: 14.32–17.32 mg alpha-acids/L		
			Decreased bitterness with good sensory acceptability				
					Alcohol content: 5.18–5.36% v/v		
			Antioxidant activity maintained almost the same percent				
Biofuel	HT	Hot trub was mixed with refuse-derived fuels (RDF) and undersized fraction from municipal solid waste (UFMSW) as bulking agents in a 3:7 ratio, bio-dried and its heating values and microbiologic composition were assessed	Using three types of waste in the process – increased sustainability	UFMSW fraction had a low bio-drying index and decreased heat values compared to RDF fraction	RDF + HT (7:3 ratio)	Use bio-drying as HT treatment for solid fuels production	(Wolny-Koładka et al., 2021)
					Bio-drying index = 9.85		
			The thermophilic temperature reached faster in the UFMSW fraction than in the RDF fraction		Lower heating values = 15,052 kJ/kg		
					UFMSW + HT (7:3 ratio)		
			RDF fraction lost 41% of its water, with high bio-drying index and heat values				
					Bio-drying index = 3.11		
					Lower heating values = 6182 kJ/kg		
Cosmetic industry	BSH	BSH extracts were prepared, TPC and antioxidant activity were assessed, applied to HaCaT and evaluated for cytotoxicity and mitochondrial activity	Good polyphenols concentration in BSH extracts, especially in ethanolic extract	BSY had better polyphenols concentrations, antioxidant activity and mitochondrial activity	TPC of aqueous extract: 7.397–8.947 mg GAE/g	Use as antiaging agent in cosmetic formulations or dietary supplements	(Censi et al., 2021)
	BSY						
			BSH extract recovered mitochondrial activity and reduced ROS formation in HaCaT cells		TPC of ethanolic extract: 13.175–15.983 mg GAE/g		
					DPPH of aqueous extract: 2.745–3.704 µmol TE/g		
					DPPH of ethanolic extract: 6.149–9.412 µmol TE/g		
Ice cream	Debittered trub	7.34% debittered trub was added to an ice cream composition and physicochemical, rheological, and textural properties were described. A sensory analysis was made	High values of fat, fiber, ash, and calorie content; high viscosity; rheological characteristics: strong gel and recoverable deformation which allow ice cream to not deform when removed from packaging; low adhesiveness, easily removed with a spoon or package; long melting time	Average sensory acceptability values than standard ice cream	Crude protein content: 7.59%	Protein-enriched ice cream	(Saraiva et al., 2020)
					Caloric value 158.06 kcal/100 g		
					Melting time: 19 min		
					Overall acceptance 5.31		

Abbreviations - BSH: spent hops, HT: hot trub, BSY: spent brewer yeast, HIPE: high internal phase emulsion, ADPH: 5′-adenosine diphosphate, HF: hexane fraction, EAF: ethyl acetate fraction, BF: butanoic fraction, AF: aqueous fraction, RDF: refuse-derived fuels, UFMSW: undersized fraction from municipal solid waste, HaCaT: human keratocyte cell line, TPC: total phenolic content, DPPH: Trolox equivalent antioxidant capacity.

### 4.1 Applications in the agriculture field

4.1.1. Animal feed. Mattioli et al. used HT and linseed as dietary supplements for rabbits and evaluated their impact on rabbit meat. The indexes of thrombogenicity (TI) slightly worsened when trub was added to the diet. The HT supplementation slightly modifies the fatty acid profile (affecting the n-6/n-3 ratio) and decreases the oxidative stability of meat lipids, but it may be effective in other animal species with high cholesterol content (pork beef, lamb) (Mattioli et al., 2020). The basal diet of pigs was supplemented with 1% BSH extract with an improvement of gain: feed ratio, lower levels of volatile fatty acids, lower counts of Streptococcus and Clostridium sp. in feces, lower intestinal expression of proinflammatory genes, and could be a sustainable solution for improving growth in pigs (Fiesel et al., 2014). Broiler chicken’s diet was supplemented with 30 mg/kg hop β-acids extract with effect upon the final meat product: prevention of myofibrillar protein oxidation and increasing redox stability due to the presence of endogenous antioxidants (anserine, carnosine, NADH, PUFA), improving the nutritional properties of chicken meat (Zawadzki et al., 2018).

4.1.2. Soil fertilizer/compost. HT and BSH may also be used as soil fertilizer, being rich in nitrogen, or as fermentation enhancers due to nitrogen, lipid, and zinc content (Hejna, 2022). Kopec et al., 2021, used HT and BSH with maize straws for composting and obtained stable compost after 60 days of intensive aerobic transformation and choosing substrates with a wide C: N ratio, HT being preferred to BSH (Kopeć et al., 2021). Craft beer producers usually discard BSH and HT as soil fertilizers for local farmers, contributing to sustainable processes (Kerby et al., 2017).

4.1.3. Crop protection. The compounds like α, β-dihydro xanthohumol, and 8-prenylnaringenin, two flavonoids found in BSH extracts, showed significant activity against methicillin-sensitive and resistant *Staphylococcus aureus* and *Staphylococcus epidermidis* while crude BSH extracts exhibited antifungal activity against *Fusaria* sp. being a potential crop-protection natural agent (Bartmańska et al., 2018).

### 4.2 Food preservatives

Hops flavonoids and their derivatives, especially with prenyl or dimethyl pyran moieties addition, showed antifeedant activity against three stored product pests: *S. granarius, T. confusum* and *T. granarium* (Jackowski et al., 2017)*.* Codina-Torella et al., 2021, evaluated BSG and BSH antioxidant capacity for food preservation. Although BSG contained ferulic acid which offered higher antioxidant values and was used for active films, BSH was also found to have good antioxidant capacity and may be a natural source for food preservation films (Codina-Torrella et al., 2021). Xanthohumol, α- or β-acids extracted from hops were assessed for their microbiological activity against food pathogens such as *Listeria monocytogenes, Staphylococcus aureus, Salmonella enterica* and *Escherichia coli* on marinated pork and meat marinade. Gram-negative bacteria (*S. enterica, E. coli*) were resistant while Gram-positive bacteria (*L. monocytogenes and S. aureus*) levels were lowered by hops extracts (mainly because of xanthohumol and β-acids). According to Kramer et al., 2015, the application of hops extracts as food preservatives is more likely to be effective in low-fat products with low pH values and at chilled storage (Kramer et al., 2015). Nionelli et al., 2018, enriched bread sourdough with hops extract which delayed the fungal growth of *Aspergillus* sp. and *Penicillium* sp. within 14 days and offered a functional product with good sensory acceptability characterized by a moderate bitterness and herbaceous consumer’s perception (Nionelli et al., 2018).

### 4.3 Dry-hopped non-alcoholic beverages

There is also potential for using BSH as a flavoring agent in the drink industry for dry-hopped non-alcoholic Beverages, especially using BSH from Cascade, Citra or Amarillo hops that impart a distinct flowery, spicy, tropical, citrus-like flavor and aroma in beer. This category includes relaxation drinks, such as hop water, hop tea, hop soda, etc. Relaxation drinks (RDs) are characterized by a mild sedative effect based, for example, on the addition of hops (Helmut Hagemann et al., 2016), acknowledged by the European Medicines Agency. The effect is at least partially due to 2-methyl-3-buten-2-ol, an oxidation product of hops' bitter compounds (Morimoto-Kobayashi et al., 2015). In addition to their sedative and stress-relieving effects, RDs may also have health-related properties, such as a favorable vitamin or mineral composition or high antioxidant levels. Growing sales of the few RDs currently available on the market, such as in Germany, indicate that there is a market for such anti-stress beverages. In addition, BHS could be used in the beverages industry as a concentrated hop extract, protein shake or high protein drinks, hop-infused honey, and hop alcoholic cocktails.

### 4.4 Medical applications

The spent hops contain the entire phytoestrogen potential of the plant, with the primary component responsible for this effect being the most potent phytoestrogen discovered to date (Bolton et al., 2020). Other notable phytoconstituents found in spent hops include desmethylxanthohumol. Because of the combination of these compounds, wasted hops can be an excellent botanical dietary supplement. Furthermore, prenylated chalcone is a common and well-studied phytoconstituent. Hops and spent hops, for example, are high in prenylated chalcones and flavonoids, both of which have shown promise in preclinical studies for cancer prevention and treatment (Venturelli et al., 2016). The natural compounds present in spent hops have antioxidant properties and the capacity to regulate carcinogen metabolism by blocking phase 1 metabolic enzymes while activating phase 2 detoxification enzymes (Karabín & Hudcov, 2016). Their ability to prevent tumor cell proliferation is very significant. Several substances in this class of secondary plant compounds have anti-tumor-initiating properties and directly inhibit cancer cell proliferation (Venturelli et al., 2016, Zanoli and Zavatti, 2008) Remarkably, they have minimal toxic effects on healthy tissues. Despite these potential pharmacological properties, challenges arise due to their limited absorption, low bioavailability, and insufficient understanding of their metabolism (Karabín and Hudcov, 2016, Venturelli et al., 2016).

On the other hand, Agnieszka Bartmańska and colleagues analyzed the antibacterial and antifungal effects of spent hops on human and plant microbial pathogens (Bartmańska et al., 2018). Flavonoid extracts exhibited significant efficacy against both methicillin-sensitive and methicillin-resistant *Staphylococcus aureus*, as well as *Staphylococcus epidermidis* strains. Antifungal activity was observed in the crude extracts of spent hops against *Fusarium oxysporum*, *F. culmorum*, and *F. semitectum*, with the lowest MIC50 value recorded at 0.5 mg/mL. The methylene chloride extract also displayed antifungal activity against Botrytis cinerea, with a minimum inhibitory concentration (MIC-50) value of 1 mg/mL. The study suggested that spent hops extracts have the potential to be effective agents for controlling economically significant plant pathogens and combating staphylococcal infections. Additionally, it has been reported that bitter acids exhibit antifungal activity against *Candida albican*s, *Trichophyton*, and *Mucor* species. They also display antibacterial activity against gram-positive bacteria, including certain species of *Micrococcus*, *Mycobacterium*, and *Streptomycetes* (Karabín and Hudcov, 2016, Zanoli and Zavatti, 2008).

Pratik Das and colleagues recently synthesized non-genotoxic, non-hemolytic organometallic silver nanoparticles using an extract of spent hops with biomedical applications (Das et al., 2022). The nanoparticles produced are spherical in shape and range in size from 10 to 50 nm. The nanoparticles demonstrated lower levels of cytotoxicity and genotoxicity towards normal cells while exhibiting excellent hemocompatibility, which are important criteria for selecting drugs. The nanoparticles obtained also showed lethality effects towards both *E. coli* and *S. aureus*, with a minimum inhibitory concentration (MIC-50) of 201.881 μg/mL and 213.189 μg/mL, respectively.

### 4.5 Cosmetic industry

Hops and hop-derived brewery by-products are rich in compounds with antioxidant, anti-inflammatory and antimicrobial properties and can be used in cosmetic formulations for anti-aging, skin-whitening, antiacne, or deodorant purposes (Pereira et al., 2022). Weber et al., 2019, developed a gel formulation with 0.3% hops extract (standardized in humulone and lupulone) with antibacterial activity against *Propionibacterium acnes* and *Staphylococcus aureus*, both pathogens involved in inflammation and acne formation (Weber et al., 2019). Dumas et al. developed a non-fragrance hops/zinc ricinolate deodorant stick with antimicrobial activity against *Corynebacterium xerosis* and *Staphylococcus epidermidis* and good odor reduction (mean malodor score dropped from 6.28 to 1.80 after 8 h of application) (Dumas et al., 2009).

## 5 Conclusions and future perspective

Hops by-products could be converted into valuable ingredients with different scopes of utilization in the food and beverages industry, agriculture, medical applications, or cosmetics.

The recovery and reuse of the hops waste in microbreweries to impart bitterness and flavor to beer, is a research direction of great interest and actuality from the perspective of the beer market, by creating new products with stylish bold aromas and flavors, as well as from the environment protection, waste management perspective, and economical view. The reuse of hops waste could provide smaller-scale brewers (who often use more energy and water to produce their product) a way to mitigate their environmental impact, while at the same time providing a new input, potentially more sustainable product that may differentiate them in a rather saturated market. In addition, could represent a sustainable alternative to food waste exploitation as an inexpensive source of valuable compounds while contributing to efficient waste reduction management and enhancing the economic potential of breweries.

Beyond the brewing industry, the hops waste could be used for food enrichment, especially for products rich in fat, and as a new source of vegetable protein. Potentially, it can be effectively used as a supplement to a culture of microorganisms in industrial bioprocesses, which yield valuable compounds for the food industry.

The disposal of waste by revalorization in an environmentally sustainable manner is an important challenge. Efficient management and further potential applications of hops waste will require sound knowledge transfer collaboration between scientists and brewers to provide sustainable results.

## Funding

This study was supported by a grant of the Romanian Ministry of Education and Research, CNCSIS-UEFISCDI, project number PN-III-P4-ID-PCE-2020-2306, within PNCDI III.

## Declaration of Competing Interest

The authors declare that they have no known competing financial interests or personal relationships that could have appeared to influence the work reported in this paper.

## Data Availability

No data was used for the research described in the article.
